# Health-Related Quality of Life of Latin-American Immigrants and Spanish-Born Attended in Spanish Primary Health Care: Socio-Demographic and Psychosocial Factors

**DOI:** 10.1371/journal.pone.0122318

**Published:** 2015-04-02

**Authors:** Miguel Ángel Salinero-Fort, Paloma Gómez-Campelo, Carmen Bragado-Alvárez, Juan Carlos Abánades-Herranz, Rodrigo Jiménez-García, Carmen de Burgos-Lunar, on behalf of the Health & Immigration Group

**Affiliations:** 1 Gerencia Adjunta de Planificación y Calidad, Atención Primaria, Servicio Madrileño de Salud, Madrid, Spain; 2 Group 27- Aging and fragility in the elderly, Hospital La Paz Institute for Health Research-IdiPAZ, Madrid, Spain; 3 Red de Investigación en Servicios de Salud en Enfermedades Crónicas (REDISSEC), Madrid, Spain; 4 Plataforma de Apoyo al Investigador Novel- PAIN Platform, Hospital La Paz Institute for Health Research-IdiPAZ, Madrid, Spain; 5 Facultad de Psicología, Universidad Complutense de Madrid, Madrid, Spain; 6 Dirección Técnica de Docencia e Investigación, Gerencia Adjunta de Planificación y Calidad, Atención Primaria, Servicio Madrileño de Salud, Madrid, Spain; 7 Departamento de Medicina Preventiva, Universidad Rey Juan Carlos, Madrid, Spain; 8 Servicio de Medicina Preventiva, Hospital Universitario La Paz, Madrid, Spain; Queensland University of Technology, AUSTRALIA

## Abstract

**Background:**

This study compares the health-related quality of life of Spanish-born and Latin American-born individuals settled in Spain. Socio-demographic and psychosocial factors associated with health-related quality of life are analyzed.

**Methods:**

A cross-sectional Primary Health Care multi center-based study of Latin American-born (n = 691) and Spanish-born (n = 903) outpatients from 15 Primary Health Care Centers (Madrid, Spain). The Medical Outcomes Study 36-Item Short Form Health Survey (SF-36) was used to assess health-related quality of life. Socio-demographic, psychosocial, and specific migration data were also collected.

**Results:**

Compared to Spanish-born participants, Latin American-born participants reported higher health-related quality of life in the physical functioning and vitality dimensions. Across the entire sample, Latin American-born participants, younger participants, men and those with high social support reported significantly higher levels of physical health. Men with higher social support and a higher income reported significantly higher mental health. When stratified by gender, data show that for men physical health was only positively associated with younger age. For women, in addition to age, social support and marital status were significantly related. Both men and women with higher social support and income had significantly better mental health. Finally, for immigrants, the physical and mental health components of health-related quality of life were not found to be significantly associated with any of the pre-migration factors or conditions of migration. Only the variable “exposure to political violence” was significantly associated with the mental health component (p = 0.014).

**Conclusions:**

The key factors to understanding HRQoL among Latin American-born immigrants settled in Spain are age, sex and social support. Therefore, strategies to maintain optimal health outcomes in these immigrant communities should include public policies on social inclusion in the host society and focus on improving social support networks in order to foster and maintain the health and HRQoL of this group.

## Background

Rapidly increasing patterns of migration have caused a growth in the number of immigrants worldwide [1–2]. In Spain, almost 12% of the population is made up of foreigners from outside the European Union [3]. Moreover, evidence suggests these immigrant groups have heterogeneous characteristics especially relating to country of origin, culture, educational level, reason for migration, and knowledge of the host country’s language [4–5].

Madrid has a population of around 6,500,000 inhabitants and a large proportion of immigrants coming from Latin America [6]. Currently, this group of immigrants accounts for 51.7% of the overall immigrant population; and this pattern is replicated in other Spanish cities [7–8]. This group is mainly composed of women, although the percentage of men is increasing. They have an average age of 34 years old, 21.2% have bachelor degrees, and are mainly employed in the service sector (53.9%) or in professional or technical jobs (12.7%) [9], and have very similar characteristics to the Latin American-born immigrants settled in the United States of America or other European countries [10].

Health Related Quality of Life (HRQoL) has been defined as an individual’s perception of their life position in the context of the culture and value systems in which they live and in relation to their goals, expectations, standards and concerns [11]. Thus, it is a multidimensional concept that includes domains such as physical health, psychological well-being, social relationships, economic circumstances, personal beliefs and an individual’s relationship with the environment.

Health outcomes and the impact of the migration process on HRQoL among Latin American immigrants have been widely analyzed. Many studies, mainly from the United States of America, highlight the so-called “healthy migrant effect” [12–14]. Recently arrived immigrants tend to be healthier than natives of the host country or at least healthier than is expected in relation to their socioeconomic characteristics. However, over time, this health advantage appears to decline. A proposed explanation of this effect is based on lifestyle and health behaviors, and on initial high social support from fellow immigrants from the same country. These factors have a protective effect on immigrants’ health, but as the acculturation process occurs these factors are progressively lost [15].

In Europe, however, previous research has reported inconsistent findings regarding health status and HRQoL of foreign-born individuals in comparison with native populations, not supporting the “healthy migrant effect”. For example, Nesterko et al. [16] reported small differences in HRQoL of immigrants and native-born Germans. Another group of studies [17–19] found that the immigrant population experienced poorer health than the native-born population. The reason for these findings is unclear, but it seems that certain immigrant populations may experience a greater burden of morbidity than the native population due to a number of psychosocial, lifestyle, and biological factors [20].

Data about the HRQoL of Latin American-born immigrants in Europe are very scarce. For example, Sundquist [21] studied a sample of Latin American refugees, and found that all immigrants had increased levels of self-rated poor health compared to natives. Sundquist concluded that being an immigrant is a risk factor for poor health and is of equal importance to more traditional risk factors such as lifestyle factors. Recently in Spain Sevillano et al. [22] have compared subjective mental and physical health among native Spaniards and immigrant groups in a cross-sectional study. The Latin American participants were from only two countries: Colombia and Bolivia (250 participants in each sub-sample). Both these studies have weaknesses in terms of their sample characteristics thus precluding the generalizability of their findings. Knowledge of Latin American-born immigrant’s health is somewhat limited in Europe; hence, this research seeks to provide new data about the HRQoL of Latin American-born immigrants in a European setting. Data for Latin American-born immigrants, from several countries and with differing legal status in the host country will be compared with that of the native population, and sociodemographic and psychosocial related variables will be considered.

The aims of this study are to compare the HRQoL of Spanish-born and Latin American-born residents in Spain, and to analyze socio-demographic and psychosocial factors associated with HRQoL. Finally, among the Latin American-born population, the possible relationship between Latin American country of origin, pre-migration factors and conditions in the host country is explored in relation to HRQoL.

## Methods

### Design

The study design and sample have been described in detail elsewhere [23]. In brief, we carried out a cross-sectional Primary Health Care multi center-based study, between January 2007 and December 2009.

Twenty primary health care centers in the northeastern area of Madrid (Spain) were invited to participate. None of these centers had specific programs aimed at immigrants. Before the study began five of these centers decided not to participate.

The northeastern area of Madrid is comprised of four districts, namely Hortaleza, Ciudad Lineal, Barajas, and San Blas. The first two have a per capita income similar to the city average, the district of Barajas has a higher income, and finally the district of San Blas has a lower income. The *Servicio Madrileño de Salud* (SERMAS) health service [24] provides universal and free health coverage, including primary, specialized and hospital health care. Immigrants should register in their area of residence in order to have access to health care, regardless of their legal status. Coverage is the same regardless of where one lives across Madrid.

### Sample

A sample was recruited from outpatients with a medical or nursing appointment at each of the centers. Thus, using a simple random selection method, 3,000 outpatients were selected from all the patients attending at each participating center. The number of patients recruited for each subgroup (Latin American-born and Spanish-born) at each center ranged between 75 and 125. The inclusion criteria were met by 2,389 patients (956 Latin American-born and 1,433 Spanish-born), who were invited to participate. A total of 1,594 participants, 903 Spanish-born and 691 Latin American-born settled in Spain, were recruited, giving a response rate of 66.7% (72.3% and 63%, respectively).

Potential participants were approached by the interviewers during primary health care visits who explained the aim of the study and invited them to participate. Eligible patients were given more details about the study in a private room, and all interviews were performed after the patient’s clinical visit. Before evaluation, the interviewer outlined the study, answered the participant’s questions, and presented the consent form for signature. Patients were evaluated at a single-time point and the results were recorded in a booklet.

The inclusion criteria were: being aged 18–55, having visited for a medical or nursing consultation, and, being a Spanish-born or Latin American-born Spanish resident. Exclusion criteria were established by the interviewers according to their clinical judgment and included: psychotic or bipolar disorder, severe chronic diseases or significant physical/ cognitive disabilities that might invalidate informed consent or interview outcomes.

### Variables

Data collection was carried out through a structured face-to-face interview delivered by two psychologists carrying out the field research. All interviewers underwent the same training on the interview methods and evaluation procedure used in the study in order to minimize interview bias.

The outcome variable was HRQoL as defined by the Spanish version of the Medical Outcomes Study 36-Item Short Form Health Survey (SF- 36) [25]. The SF-36 contains 36 items, covering eight health domains: physical functioning, role limitations due to physical health problems, bodily pain, general health, vitality, social functioning, role limitations due to emotional problems and mental health; which contribute to a composite physical health (PCS; Physical Component Summary) and mental health (MCS; Mental Component Summary) components summary. Scores were transformed from 0 to 100; and higher scores indicate greater quality of life [26]. In the study, Cronbach´s Alpha (α) for the subcales were reported to range from 0.80 to 0.97 (physical functioning, α = 0.90; role limitations due to physical health problems α = 0.97; bodily pain α = 0.80; general health α = 0.80; vitality α = 0.83; social functioning α = 0.82; role limitations due to emotional problems α = 0.95; and mental health α = 0.83).

Social support was assessed using the Spanish version of the Medical Outcomes Study-Social Support Survey (MOS-SSS) [27–28]. This is a self-administered survey comprised of 19 items that include a global dimension for social support and four sub-dimensions: emotional/informational; positive social interaction; affective support; and tangible or instrumental support. The total score was transformed into a 0–100 scale, with higher scores indicating high social support [27]. α for the total scale was 0.97.

Additionally, sociodemographic variables were obtained through an *ad hoc* questionnaire including the following: gender, age, geographic origin (Spanish-born vs. Latin American-born Spanish resident), marital status (single, divorced or widowed vs. married or cohabiting), educational level (with four standard hierarchical levels: no education completed, primary, secondary, and university), occupational status (management position, administrative officer/ self employed/ supervisor, manual skilled worker/ unskilled worker, unemployed), income level (monthly income in three categories: less than 500 Euros, 500–1000 Euros, more than 1000 Euros), and length of residence in Spain (always for Spanish-born participants, and for the Latin American-born: less than five years, and five or more years of residence).

Additionally, we collected some specific data about pre-migration factors from the Latin American-born participants: Latin American country of origin, legal status in the host country (illegal: undocumented residents in Spain; legal: documented residents in Spain) exposure to different types of violence (yes vs. no), reasons for migration (economics vs. others) and conditions for migration (alone vs. with someone).

### Ethical Approval

This study was approved by the local research ethics committee (Ramón y Cajal Hospital, Madrid), and an informed consent form was signed by all participants.

### Statistical analysis

Descriptive statistical analysis was calculated for all variables. Quantitative variables were summarized as mean and standard deviation (SD), and categorical variables summarized by frequency and percentage.

Error-graphs were plotted to analyze differences in PCS and MCS stratified by length of residence in Spain, and by gender.

Firstly, Pearson’s correlation test or Spearmen rank’s correlation was performed to evaluate the unadjusted association among HRQoL (PCS and MCS) and socio-demographic and psychosocial factors. Variables that were found to have statistical significance p<0.20 in the univariate analysis, and considered clinically relevant in accordance with previous literature, such as age and gender, were included in the adjusted analyses.

Secondly, hierarchical regression analysis of adjusted models was carried out in the following manner: in step one, geographic origin was included as a predictor, taking the Latin American-born participants as the reference group. In steps two, three, and four, age, gender, and the remaining variables (global social support, marital status and monthly income) were included. Regression analyses were conducted for the entire sample (N = 1,594) as well as stratified by gender, and stratified by geographic origin. When stratified by gender, geographic origin was included in step one, with age and the remaining variables included subsequently. Finally, when stratified by geographic origin, age was included in step one, gender in step two, and the remaining variables were included afterwards.

Finally, to examine the possible relationship between pre-migration factors and HRQoL (PCS and MCS) among Latin American-born participants the Student’s t-test was applied.

In all cases, the accepted level of significance was 0.05 or less, with a 95% Confidence Interval (95% CI). The statistical analysis was performed using the Statistical Package for Social Sciences (SPSS for Windows, version 19.0; IBM Corp, Armonk, New York, USA).

## Results

### Sample characteristics

The demographic characteristics of participants and statistically significant differences between the groups are presented in Table 1. Latin American-born participants were younger, had a lower educational level, worked in less skilled jobs, and had a lower monthly-income than the Spanish-born participants. Spanish-born participants had a significantly larger social support network (approximately 9 people) compared to those in the Latin-American-born group (6 people). Furthermore, Spanish-born participants reported better social support than Latin American-born immigrants in the global scale and in all subscales (all p-values<0.05).

**Table 1:** Distribution of socio-demographic variables and social support in the study population, stratified by country of origin and gender.

	**Overall**			**Latin American-born**			**Spanish-born**		
	**N = 1,594**	Men	Women	**Overall**	Men	Women	**Overall**	Men	Women
		(n = 531)	(n = 1,063)	**(n = 691)**	(n = 278)	(n = 413)	**(n = 903)**	(n = 253)	(n = 650)
**Age** (years), mean (SD) [a,c,d,f]	**35.9 (10.7)**	34.2 (9.7)	36.8 (11.1)	**34.4 (9.7)**	33.8 (9.4)	34.9 (9.9)	**37.1 (11.3)**	34.6 (10.1)	38.1 (11.6)
**Age group** (years), % (n) [a,c,d,f]									
18–29	**31.8 (507)**	35.6 (189)	29.9 (318)	**34 (235)**	36.7 (102)	32.2 (133)	**30.1 (272)**	34.4 (87)	28.5 (185)
30–39	**35 (558)**	39 (207)	33 (351)	**39.4 (272)**	38.8 (108)	39.7(164)	**31.7 (286)**	39.1 (99)	28.8 (187)
40–55	**33.2 (529)**	25.4 (135)	37.1 (394)	**26.6 (184)**	24.5 (68)	28.1 (116)	**38.2 (345)**	26.5 (67)	42.8 (278)
**Marital status,** % (n) [c,e,f]									
Married/ Cohabiting	**50.8 (810)**	47.1 (250)	52.7 (560)	**51.9 (358)**	52 (144)	51.8 (214)	**50.1 (452)**	41.9 (106)	53.2 (346)
**Children,**									
Yes % (n) [a,c,d,e]	**54.1 (706)**	59.5 (316)	63.9 (679)	**76 (525)**	72.3 (201)	78.5 (324)	**52 (470)**	45.5 (115)	54.6 (355)
Number, mean (SD) [a,c,d,e,f]	**1.06 (1.26)**	0.9 (1.2)	1.1 (1.3)	**1.4 (1.4)**	1.3 (1.3)	1.5 (1.6)	**0.8 (1.1)**	0.6 (0.9)	0.9 (1)
**Educational level, %** (n) [a,c,d,e]									
No education completed	**0.6 (10)**	0.4 (2)	0.8 (8)	**0.9 (6)**	0.7 (2)	1 (4)	**0.4 (4)**	0 (0)	0.6 (4)
Primary	**29.4 (468)**	29.2 (155)	29.4 (313)	**31.9 (220)**	35.7 (99)	29.3 (121)	**27.5 (248)**	22.1 (56)	29.5 (192)
Secondary	**32.6 (519)**	34.7 (184)	31.5 (335)	**40.1 (277)**	37.9 (105)	41.6 (172)	**26.8 (242)**	31.2 (79)	25.1 (163)
University	**37.4 (596)**	35.6 (189)	38.3 (407)	**27.1 (187)**	25.6 (71)	28.1 (116)	**45.3 (409)**	46.6 (118)	44.8 (291)
**Occupational status, %** (n) [a,b,c,d,e,f]									
Manager	**8.5 (124)**	12.6 (50)	7 (74)	**1.4 (8)**	3.5 (5)	0.7 (3)	**12.8 (116)**	17.8 (45)	10.9 (71)
Administrative/Self-employed	**25.1 (366)**	24.2 (96)	25.4 (270)	**12.2 (68)**	12.6 (18)	12.1 (50)	**33 (298)**	30.8 (78)	33.8 (220)
Manual worker	**42 (613)**	48.5 (192)	39.6 (421)	**66.2 (368)**	68.5 (98)	65.4 (270)	**27.1 (245)**	37.2 (94)	23.2 (151)
Unemployed	**24.4 (356)**	14.6 (58)	28 (298)	**20.1 (112)**	15.4 (22)	21.8 (90)	**27 (244)**	14.2 (36)	32 (208)
**Monthly income, %** (n) [a,b,c,d,e,f]									
< 500 Euros	**6.6 (106)**	4.9 (26)	7.5 (80)	**11.4 (79)**	7.6 (21)	14 (58)	**3 (27)**	2 (5)	3.4 (22)
500–1000 Euros	**34 (542)**	32.2 (171)	34.9 (371)	**53 (366)**	51.4 (143)	54 (223)	**19.5 (176)**	11.1 (28)	22.8 (148)
> 1000 Euros	**59.4 (946)**	62.9 (334)	57.6 (612)	**35.6 (246)**	41 (114)	32 (132)	**77.5 (700)**	87 (220)	73.8 (480)
**Social support,** mean (SD)									
Network size [a,d,e]	**6.80 (6)**	7.6 (7.1)	7.7 (6.8)	**6.1 (6.6)**	6.3 (6)	6 (6.7)	**8.9 (6.9)**	9.1 (7.4)	8.8 (6.7)
Global [a,b,d,e]	**75.75 (23.7)**	75.8 (24.7)	75.7 (23.2)	**68.2 (26.6)**	71.6 (27.5)	66 (25.8)	**81.5 (19.5)**	80.5 (20.4)	81.9 (19.1)
Emotional/ Informational [a,b,d,e]	**74.86 (25.3)**	74.4 (25.9)	75.1 (25.0)	**67.1 (28.3)**	70.1 (28.6)	65.1 (27.9)	**80.8 (21)**	79.0 (21.9)	81.5 (21.4)
Positive social interaction [a,b,d,e]	**76.59 (25.3)**	76.8 (25.5)	76.5 (25.2)	**69.9 (28.2)**	73.1 (27.9)	67.8 (28.2)	**81.7 (21.6)**	80.9 (22.0)	81.9 (1.4)
Affective [a,c,d,e,f]	**82.48 (24.6)**	80.4 (26.4)	83.5 (23.6)	**77 (27.7)**	77.8 (28.6)	76.5 (27.1)	**86.7 (21)**	83.3 (23.5)	88 (19.9)
Instrumental [a,b,d,e,f]	**71.63 (29.5)**	74.4 (29.2)	70.3 (29.5)	**62.3 (32.3)**	68.6 (32.2)	57.9 (31.7)	**78.8 (24.9)**	80.7 (24.1)	78.1 (25.1)

a: Differences between Spanish-born and Latin American-born p-value < 0.05.

b: Differences between Latin American-born men and women p-value < 0.05.

c: Differences between Spanish-born men and women p-value < 0.05.

d: Differences between Latin American-born and Spanish-born women p-value < 0.05.

e: Differences between Latin American-born and Spanish-born men p-value < 0.05.

f: Differences between women and men.

Bold type indicates total data.

Nearly half of the Latin American-born participants were Ecuadorean (43.3%), 15.3% were Peruvian, 14.5% Colombian, and the rest (26.9%) came from other Latin American countries. Before arriving to Spain, 39.6% of Latin American-born participants held administrative positions or were self-employed, 31.9% were manual workers, 22.9% were unemployed and 6% were managers. The most frequently cited reasons for migration were economic factors, family reunification and the search for freedom or new challenges (65.1%, 12.4%, and 12.2%, respectively). Furthermore, 69% migrated alone, 7.5% migrated with a partner but without children and the remainder of this group migrated along with other family members. 86.4% were legally allowed to live in Spain, either having residency status and/ or a work permit or dual Spanish nationality, with 23.1% reporting having dual Spanish nationality. The mean length of residence in Spain was 6.5 years (SD = 4.6 years), and 41.9% of Latin American-born participants had stayed for more than five years. Being a victim of political violence was reported by 5.8% of these participants, and 7.8% cited they migrated due to family violence in their country. Finally, the loss rate for the variable “length of residence in Spain (years)” was 19.7%.

### Health Related Quality of Life: Spanish-born versus Latin American-born

The mean scores for the SF-36 subscales, stratified by gender and geographic origin, are presented in Table 2. Physical functioning was the highest score among the eight health domains (90.8, SD = 16.7) and vitality was the lowest (58.5, SD = 21).

**Table 2:** Descriptive data on the SF-36 mean scores for each subscale, stratified by gender and country of origin.

	**Overall**			**Latin American-born**			**Spanish-born**		
	**N = 1,594**	Men	Women	**Overall**	Men	Women	**Overall**	Men	Women
		(n = 531)	(n = 1,063)	**(n = 691)**	(n = 278)	(n = 413)	**(n = 903)**	(n = 253)	(n = 650)
**Physical functioning** [a,b,c,f]	**90.8 (16.7)**	94.2 (14.8)	89.1 (17.3)	**92.4 (15.5)**	95.5 (13)	90.1 (16.8)	**89.7 (17.4)**	92.9 (16.6)	88.6 (17.5)
**Role limitations due to physical health problems** [b,c,f]	**70.5 (43.6)**	77.4 (39.3)	67.2 (45.1)	**71.9 (43.4)**	77.2 (40.3)	68 (45.3)	**69.6 (43.6)**	77.6 (38.3)	66.8 (45.0)
**Role limitations due to emotional problems** [b,c,f]	**77.1 (40.1)**	85.5 (33.5)	73.1 (42.2)	**77 (40.5)**	85.1 (34.4)	70.9 (43.6)	**77.2 (39.8)**	86.1 (32.5)	74.2 (41.5)
**Vitality** [a,b,c,f]	**58.5 (21)**	66.4 (19.1)	54.7 (20.8)	**60.3 (20.6)**	66.7 (19.5)	55.6 (20.1)	**57.3 (21.2)**	66.1 (18.7)	54.3 (21.2)
**Mental health** [b,c,d,f]	**63.4 (21.3)**	70.6 (20.3)	60 (21)	**62.1 (21.7)**	68.8 (21.1)	57.1 (20.8)	**64.3 (21.1)**	72.6 (19.3)	61.5 (21)
**Social functioning** [b,c,f]	**79.6 (25.6)**	84.1 (22.4)	77.5 (26.7)	**80.9 (24.3)**	84.9 (21.2)	77.9 (26)	**78.8 (26.4)**	83.3 (23.6)	77.3 (27.1)
**Bodily pain** [b,c,f]	**65.3 (27.8)**	74.5 (26)	61 (27.5)	**67.1 (27.7)**	76.9 (25)	59.8 (27.4)	**64.2 (27.8)**	71.9 (26.8)	61.6 (27.6)
**General health** [b,c,f]	**68.7 (20.6)**	71.5 (19.7)	67.3 (21)	**65.8 (20.4)**	71.7 (19.9)	66.2 (20.5)	**68.7 (20.8)**	71.3 (19.5)	67.9 (21.2)
**Physical Component Summary** [a,b,c,f]	**49.9 (9.1)**	51.4 (8.6)	49.3 (9.2)	**50.77 (8.9)**	52.1 (8.4)	49.8 (9.2)	**49.4 (9.1)**	50.5 (8.8)	49 (9.2)
**Mental Component Summary** [b,c,f]	**43.9 (12.9)**	47.5 (11.6)	42.3 (13.2)	**43.6 (12.8)**	46.8 (11.8)	41.2 (13)	**44.1 (13.1)**	48.2 (11.5)	42.8 (13.3)
**Physical Component Summary** (% above mean) [b,c,f]	**60.2**	69.9	55.7	**63**	73.1	55.6	**58.4**	66.3	55.7
**Mental Component Summary** (% above mean) [b,c,f]	**39.8**	53.1	33.6	**37.7**	48.9	29.5	**41.2**	57.8	35.6

a: Differences between Spanish-born and Latin American-born p-value < 0.05.

b: Differences between Latin American-born men and women p-value < 0.05.

c: Differences between Spanish-born men and women p-value < 0.05.

d: Differences between Latin American-born and Spanish-born women p-value < 0.05.

e: Differences between Latin American-born and Spanish-born men p-value < 0.05.

f: Differences between women and men.

Bold type indicates total data.

The physical component summary was significantly higher than the mental component summary for all participants whether data was non-stratified or stratified by gender (all p-value <0.001, except for Spanish-born men p = 0.037). When stratifying by geographic origin, there were significant differences in PCS, physical functioning and vitality, with higher scores in the Latin American-born group. Also, men showed significantly better subjective health in all the scales compared to women, both in the overall sample and within the Latin American-born and Spanish-born populations. When stratifying by geographic origin, there were significant differences in PCS, physical functioning and vitality, with higher scores in the Latin American-born group. Also, data reported significant gender differences in the eight subscales of HRQoL, for the overall sample and for the two geographical areas of origin.

**Fig 1:**
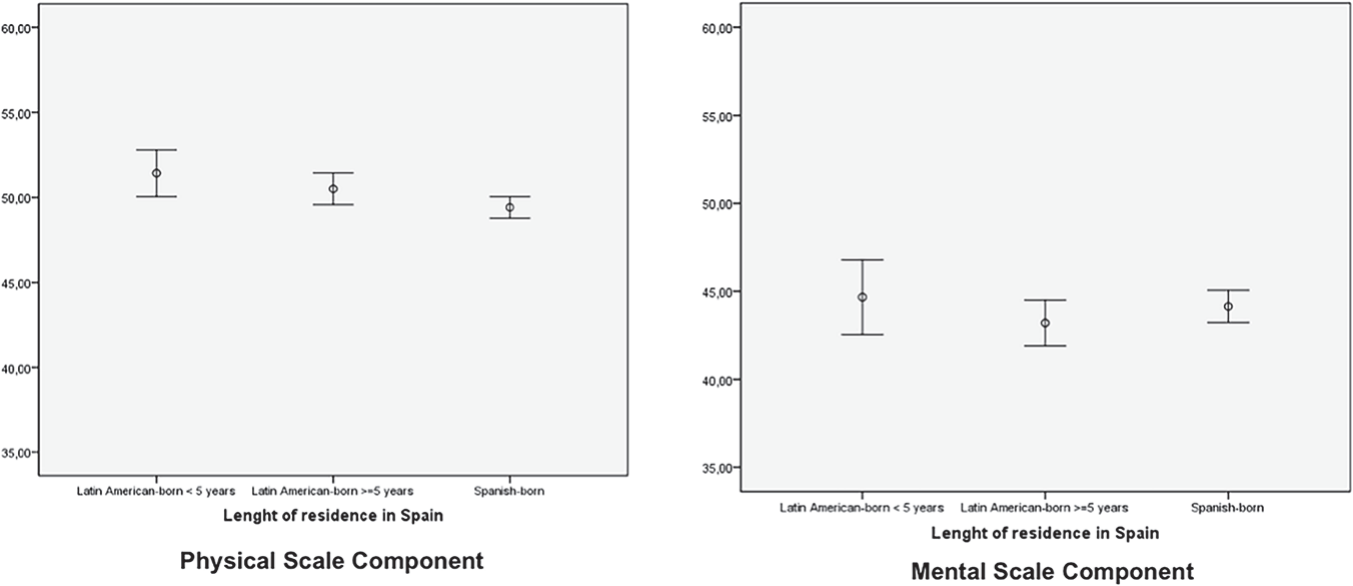
Physical and Mental components of Health Related Quality of Life, stratified by length of residence in Spain.

**Fig 2:**
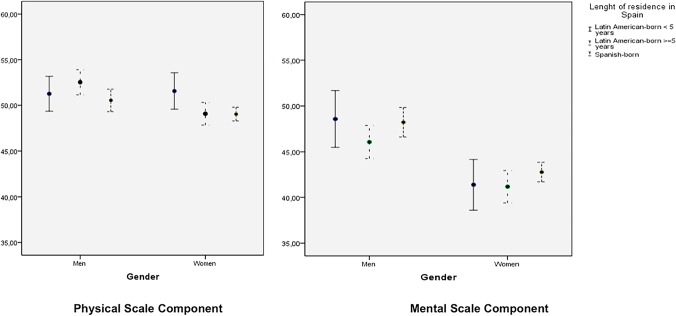
Physical and Mental components of Health Related Quality of Life, stratified by length of residence in Spain and by gender.

Fig 1 shows the differences in PCS and MCS scores between the three groups (Latin-American born with <5 years of residence in Spain, Latin-American born with ≥ 5 years in Spain, and Spanish born) and Fig 2 shows the same data stratified by gender. There were no statistically significant differences between the three groups (Fig 1), however, Latin American-born men with five or more years of residence reported significantly higher PCS than women in this group (Fig 2). Furthermore, significant differences were noted in MCS when stratifying by gender and by length of residence in Spain. Finally, Spanish-born women reported significantly worse MCS than the three groups of men. Furthermore, Latin American-born women with five or more years of residence in Spain reported worse MCS than their male counterparts, independently of the length of residence in Spain.

### Health related Quality of Life and related factors

Table 3 summarizes the association, crude and adjusted, between: socio-demographic characteristics, psychosocial factors, and MCS and PCS respectively, for the entire sample, and stratified by gender.

**Table 3:** Hierarchical regression analysis for variables predicting Physical Scale Component and Mental Scale Component, stratified by gender.

	**Variable entered**	**crude** *b* ^*a*^		*b* ^*a*^				**R** ^2^	**Model F**
				**Step 1**	**Step 2**	**Step 3**	**Step 4**		
**ENTIRE SAMPLE** (N = 1,594)									
**Physical component summary**	B_0_			50.77	54.95	57.16	54.39	0.035	8.977**
	Geographic origin^b^		0.09**	0.07**	0.06*	0.04	0.06*		
	Age		-0.16**		-0.15**	-0.14**	-0.11**		
	Gender^c^		0.11**			0.08**	0.08**		
	Social support		0.08**				0.08**		
	Marital status^d^		-0.07*				-0.05		
	Monthly income^e^		-0.02				-0.01		
**Mental component summary**	B_0_			43.61	46.82	54.32	34.00	0.166	44.181**
	Geographic origin		-0.03	-0.02	-0.03	-0.06*	0.05		
	Age		-0.08**		-0.08**	-0.06*	-0.01		
	Gender		0.19**			0.19**	0.19**		
	Social support		0.35**				0.34**		
	Marital status		0.08**				0.04		
	Monthly income		0.14**				0.09**		
**MEN** (N = 531)									
**Physical component summary**	B_0_			50.54	55.98	53.75		0.038	4.257**
	Geographic origin^b^		0.10*	1.59	1.45	1.61			
	Age		-0.19**		-0.16**	-0.16***			
	Social support		0.09			0.02			
	Marital status^d^		0.05			0.68			
	Monthly income^e^		-0.01			0.18			
**Mental component summary**	B_0_			48.23	50.26	29.05		0.131	13.572**
	Geographic origin		-0.08	-1.38	-1.44	0.94			
	Age		-0.05		-0.06	0.03			
	Social support		0.36**			0.18**			
	Marital status		0.04			-0.61			
	Monthly income		0.14**			2.11*			
**WOMEN** (N = 1,063)									
**Physical component summary**	B_0_			49.04	52.74	49.45		0.20	4.678**
	Geographic origin^b^		0.04	0.72	0.46	0.92			
	Age		-0.12**		-0.97**	-0.07*			
	Social support (global scale)		0.07*			0.04*			
	Marital status^d^		-0.10**			-1.48*			
	Monthly income^e^		-0.03			0.08			
**Mental component summary**	B_0_			42.77	45.40	20.97		0.138	29.372**
	Geographic origin		-0.06	-1.54	-1.73	1.67			
	Age		-0.05		-0.07	-0.01			
	Social support (global scale)		0.36**			0.22**			
	Marital status		0.12**			1.77			
	Monthly income		0.14**			1.81*			

*b* ^a:^ standardized coefficients except for constant term.

^b^: Geographic origin: dummy coded; Spanish-born as reference category (0).

^c^: Gender: dummy coded; women as reference category (0)

^d^:Marital status: dummy coded; single as reference category (0).

^e^: Monthly income: dummy coded; <1000 euros as reference category.

*p<0.05

** p<0.01

Across the entire sample, the adjusted model shows that Latin American-born participants, younger participants, men, and those with higher perceived social support, reported significantly higher physical health scores. Men with higher perceived social support and higher monthly income reported significantly better mental health.

Hierarchical regression analysis stratified by gender shows that for men physical health was only positively associated with younger age, whereas for women, in addition to age, social support and marital status were significantly related. Both men and women with higher income and higher social support, experienced significantly better mental health.

Regression analyses for both Latin American-born immigrants and Spanish-born participants are shown in S1 Table and S2 Table. These show very similar results to those of the entire sample.

**Fig 3:**
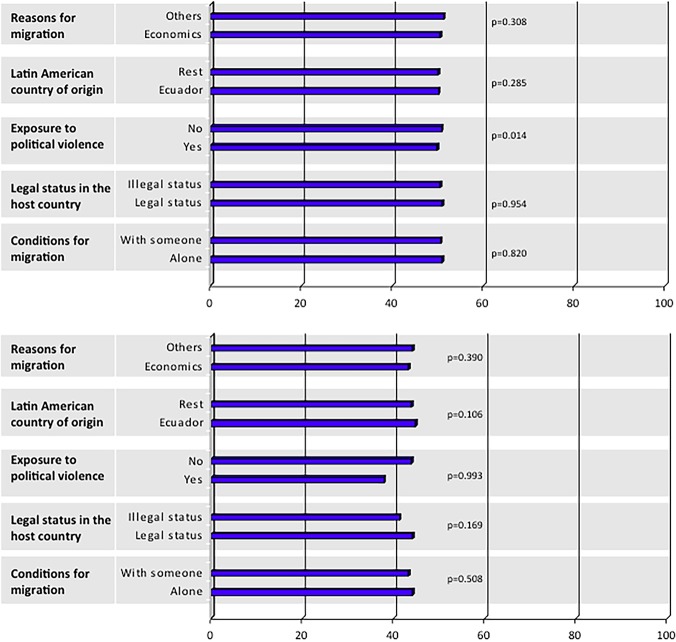
Physical (A) and mental (B) components of Health Related Quality of Life and pre-migration factors for Latin American-born immigrants.

Finally, no relationship between pre-migration factors and PCS (Fig 3A) and MCS (Fig 3B) was found, except for PCS and exposure to political violence (p = 0.014), which suggests that Latin American-born participants not exposed to political violence in their country of origin showed significantly better physical health than those exposed to it.

## Discussion

### Health Related Quality of Life: Spanish-born versus Latin American-born

In general, our findings suggest small and not statistically significant differences in HRQoL between Latin American-born immigrants settled in Spain and Spanish-born residents. IA possible explanation could be that immigrants in our sample share the same language (Spanish) with the Spanish-born participants, which could be a protective factor throughout the migration process. Our findings are consistent with previously reported data from some European studies with non-Latin American-born immigrants [29], confirming better HRQoL in immigrants compared to participants native to the host country. Several European studies have also found differences in the subjective physical health component [16,30] with native populations faring better. For example, Sundquist [21] conducted a study with face-to-face interviews of 338 Latin American refugees, 60 Latin American migrants and 1,159 Swedes; concluding that immigrants had increased self-rated poor health compared to Swedes. In this study, there was a big difference between the immigrants and natives in terms of language. Thus, unfamiliarity with the language may hinder integration into a host country, and worsen both physical and mental HRQoL.

The cultural similarity between both groups in our study sample could be associated with a fewer barriers in the migration process that may explain the absence of differences in HRQoL. However, to our knowledge, very few Spanish studies have analyzed this issue, thus reducing our ability to compare results. Age could also be a significant factor. In the present study, the immigrant participants were slightly younger (34.4 years) than the Spanish-born (37.1 years). This could be an explanation for the higher physical HRQoL scores seen in Latin American immigrants. A possible explanation for these differences could be that the Swedish study analyzed a sample of Latin American refugees, whilst our sample included Latin American-born individuals with a variety of legal statuses but non with refugee status.

Unexpectedly, Latin American-born immigrants were found to be physically healthier than their Spanish-born counterparts in several aspects such as physical functioning, vitality and PCS. This relative physical health advantage experienced by Latin American-born participants has also been found in other Spanish based studies. García-Gómez and Oliva [5] compared data from the Cataluña Health Survey (Spain), and argued that immigrants were less likely to report bad physical health but were more likely to report bad mental health levels compared to Spanish born participants. Also, their data indicate that foreign immigrants from poor countries had the poorest socio-economic status, but relatively better health, especially among men with a short length of residence. Similarly, a recent paper [22] comparing immigrants to Spain from Colombia, Bolivia, Romania, Morocco and Sub-Saharan Africa to native Spaniards, also partially supports these findings. This research suggests that, after controlling for age, socio-economic and marital status, male immigrants from Colombia and Sub-Saharan Africa reported better physical health than natives. Except for Colombians, immigrants had poorer mental health than natives, especially Sub-Saharan African men and Bolivian women.

Thus, in Spain, the healthy migrant effect could be attributed to the fact that most immigrants are healthy young people who have come to work and that disease occurrence is related to their assimilation of Western lifestyles, working conditions and their socio-familial context [31]. Therefore, data from this study on the mental health component of HRQoL is in line with other studies [32] that suggest no differences between the two populations in subjective mental health.

### Health related Quality of Life and related factors

Our data indicate that being male is a predictor for better physical and mental health. Among women high social support is a predictor for better physical and mental health. Among men, high social support is only a predictor for better mental health. Previous research confirms the advantage of being male in subjective health results [33]; for example, Borrell et al. [34] illustrate that women in lower socio-economic groups have the worst indicators for morbidity and self-assessed health. The effect of social relationships on health is important, for example, data indicate a 50% increased likelihood of survival for participants with stronger social relationships [35]. A study carried out in the Basque Country (Spain) suggests that low social support was related to poor HRQoL; identifying the key role of social support in understanding health inequalities among immigrants [36] which is in agreement with our findings. In addition, our data suggest that subjective mental health is not dependent on geographical origin, whereas being Latin American-born plays an important role in having better physical health.

In our study sample, young participants were found to be physically healthier; this remained the case when results were stratified by gender. As Daher et al. suggest [33], this may be explained by the fact that younger participants experience lower morbidity due to their ability to better adapt to new environmental conditions.

In accordance with previous studies [29], our study found a low monthly income is associated with lower HRQoL (MCS) for both men and women. However, there was no association with PCS. Also, an interaction between gender and income was identified. These findings are partly consistent with previous Spanish studies [31] that support the healthy immigrant effect. These have found that immigrants, especially men with a shorter length of residence, had the poorest socio-economic status but relatively better health. Therefore, it seems that HRQoL cannot be understood independently of age, gender, socio-demographic status, social support and geographical origin.

It is of interest to explore the possible HRQoL score differences between groups according to their length of residence in Spain, geographical origin and gender. Our results did not reflect data from previous studies [37] as none of these factors were seen to have a significant relationship with subjective physical and mental health. Length of residence in the host country was not seen to be relevant but, gender may be an important variable to consider.

Thus, Latin American-born women with five or more years of residence in Spain reported worse MCS than their male counterparts with any length of residence in Spain. As mentioned above, our findings are in keeping with some previous research [38] but do not support the idea of a “healthy migrant effect” [12–14]. One possible explanation for this may be the higher monthly incomes and better perceived social support experienced by Latin American-born immigrants with a longer length of residence in Spain compared to immigrants with a shorter length of residence. Thus, contrary to expected trends, immigrants with longer residency had better socio-economic conditions than recent immigrants. However, no data are available on the possible changes to their economic and social situation since their time of arrival in the host country. Previous Spanish studies have highlighted the knowledge gap on the association between length of residence in the host country and HRQoL, and there are no national data available to compare our findings with.

Previous studies [38] have addressed the association between political violence experienced before emigrating and mental health issues and HRQoL. The divergence between these data and our findings may be attributed to differences in the study samples. In the Eisenman et al. study [39], 54% of immigrants from Latin America experienced political violence in their home country. Most of the immigrants in this study came from the following countries: Mexico (41.5%), El Salvador (32.5%) and Guatemala (17.7%). In our study sample there was a low prevalence of exposure to political violence before immigration to Spain. Only 5.8% of participants had experienced this and they were largely from Ecuador (43.3%) and Peru (14.5%). Finally, to our knowledge, there are no official data on exposure to political violence experienced by Latin American-born immigrants in Spain; consequently it is not possible to compare our results to other findings.

This study has several limitations. The study sample was not population based, and a cross-sectional approach with a sample composed of outpatients visiting Primary Health Care Centers was used. This may not be representative of all Latin American-born immigrants settled in Spain or of the Spanish-born community. Moreover, at the time of the study, despite the fact that the entire resident population in Spain had free health coverage for attendance or follow-up, differences may be found between the two groups due to differences in the use of the healthcare service. Soler-González et al. [40] studied a population attending Primary Care services in Spain and showed that immigrants have a significantly higher probability of attending appointments compared to the native community (3 or more visits vs. 1 or 2 visits). Also, Fuertes et al. [41] have recently found that there is no association between the reason for the visit and nationality when exploring visits to primary health care centers in Spain. The most common reasons for visits to primary health care facilities cited by the Latin American-born and the Spanish-born participants included: respiratory, musculoskeletal and digestive diseases. Therefore, it seems that being an immigrant in Spain is not a barrier to accessing the health care system, and that the reasons for the visits do not differ between these populations.

Another limitation is the lack of data on primary health care center attendance or previous personal medical history. However, recent data from Spain [32] estimate the prevalence of selected chronic illnesses based on electronic clinical records in primary care settings. This data show a lower prevalence among the immigrant population. Therefore, the authors believe that if any selection bias exists it is probably small and does not significantly affect the study results.

Additionally, the high number of female participants in this study should be noted. This is due to women’s increased use of primary health care services compared to men [42].

The cross-sectional design of this study limits the possibility of establishing causal relationships between variables, and the findings should be carefully interpreted. Moreover, existing literature [39] suggests that it is important to disaggregate health data by country of origin. This study only focuses on Latin American-born immigrants. Nevertheless, the immigrants in our sample possess very similar characteristics (sex, age, educational level and occupational status) to that of Latin American-born immigrants in other European and North-American cities [9–10], and to non-Latin American-born immigrants in other Spanish studies [5,36]. This may allow us to carefully generalize our findings to other immigrant populations, in and out of Spain, albeit with caution.

Finally, the willingness to participate in health survey studies according to several variables, such as ethnicity, must be considered as a potential bias of this type of research. However, recent studies found very small differences in the willingness of minorities to participate in health research compared to non-minority individuals [43], suggesting that racial and ethnic minorities are as willing as non-minority individuals to participate in health research.

Despite the limitations, our findings suggest that there are small differences in HRQoL between Latin American-born immigrants settled in Spain and Spanish-born residents. Latin American-born immigrants who have settled in Spain show better subjective physical health compared to Spanish-born residents. Also, there is a clear variation in HRQoL by geographical origin, age, gender, social support and income. Therefore, Latin American-born participants, younger participants, men, and those with higher perceived social support, reported better physical health. Furthermore, younger participants, with higher perceived social support and higher monthly income, reported better mental health. Interestingly, immigration-related factors were not related to HRQoL.

Further studies are needed to clarify HRQoL differences between the Latin American-born immigrants and the native population, and studies that include other foreign-born groups are required. In Spain, data between 2006 and 2010 show that [44–45] the recession has significantly increased the frequency of mental health disorders and alcohol abuse among primary care attendees, particularly among families experiencing unemployment and mortgage and loan payment difficulties. Thus, given the known relationship between mental health and quality of life, we should consider that further research on HRQoL and health of immigrants is necessary to analyze and characterize the current situation in light of the international economic crisis. Additionally, further in-depth studies in Spain exploring the association between health and quality of life, according to country of origin and place of residence are still needed and comparisons with other European countries should also be made.

Despite its limitations, this research offers an insight into the HRQoL of Latin American-born immigrants settled in Spain. It seems that immigrants report better subjective physical health-related quality of life compared to Spanish-born individuals. Also, Latin American-born participants, younger people, men, and those with higher perceived social support, reported higher physical health. Furthermore, younger participants, with higher perceived social support and higher income, reported higher mental health scores. Interestingly, exposure to political violence was the only immigration factor related to the mental component of health-related quality of life.

Finally, the key factors to understanding HRQoL among Latin American-born immigrants settled in Spain are age, sex and social support. Therefore, strategies to maintain optimal health outcomes in these immigrant communities should include public policies on social inclusion in the host society and focus on improving social support networks in order to foster and maintain the health and HRQoL of this group.

## Supporting Information

S1 Table Hierarchical regression analysis for variables predicting Physical Scale Component and Mental Scale Component for Latin American-born (N = 691). (DOC)

S2 Table Hierarchical regression analysis for variables predicting Physical Scale Component and Mental Scale Component for Spanish-born (N = 903). (DOC)
