# Supplementary material for: Health-Related Quality of Life of Latin-American Immigrants and Spanish-Born Attended in Spanish Primary Health Care: Socio-Demographic and Psychosocial Factors
Source: PLoS One. 2015 Apr 2;10(4):e0122318. doi: 10.1371/journal.pone.0122318 (PMC4383449; doi:10.1371/journal.pone.0122318)
Supplement: S2 Table — (DOC) [file pone.0122318.s002.doc]

S2 Table. Hierarchical regression analysis for variables predicting Physical Scale Component and Mental Scale Component for Spanish-born (N=903).

| **Variable entered** | **crude *b*^a^** | ***b*^a^** | | | **R^2^** | | **Model F** |
| --- | --- | --- | --- | --- | --- | --- | --- |
|  |  | **Step 1** | **Step 2** | **Step 3** |  |  |  |
| **Physical Scale Component** |  |  |  |  |  |  |  |
| B_0_ |  | 54.47 | 54.02 | 48.05 | 0.039 |  | 7.427*** |
| Age | -0.17** | -0.17*** | -0.17*** | -0.12** |  |  |  |
| Gender^c^ | 0.07* |  | 0.05 | 0.04 |  |  |  |
| Social support (global scale) | 0.11** |  |  | 0.10** |  |  |  |
| Marital status^d^ | -0.03 |  |  | -0.81* |  |  |  |
| Monthly income^e^ | -0.03 |  |  | 0.04 |  |  |  |
|  |  |  |  |  |  |  |  |
| **Mental Scale Component** |  |  |  |  |  |  |  |
| B_0_ |  | 46.23 | 43.87 | 19.21 | 0.152 |  | 29.294*** |
| Age | -0.05 | -0.05 | -0.03 | 0.01 |  |  |  |
| Gender | 0.18** |  | 0.18*** | 0.18*** |  |  |  |
| Social support (global scale) | 0.33** |  |  | 0.31*** |  |  |  |
| Marital status | 0.06 |  |  | 0.06 |  |  |  |
| Monthly income | 0.16** |  |  | 0.09** |  |  |  |
|  |  |  |  |  |  |  |  |

*b*^a^: standardized coefficients except for constant term.

^b^: Country of origin: dummy coded; Spanish-born as reference category (0).

^c^: Gender: dummy coded; women as reference category (0)

^d^:Marital status: dummy coded; single as reference category (0) and Married/ Cohabiting (1).

^e^: Monthly income: dummy coded; <1000 euros as reference category.

*p<0.05

** p<0.01

*** p<0.001
